# Neural networks for modeling gene-gene interactions in association studies

**DOI:** 10.1186/1471-2156-10-87

**Published:** 2009-12-23

**Authors:** Frauke Günther, Nina Wawro, Karin Bammann

**Affiliations:** 1 University of Bremen, Bremen Institute for Prevention Research and Social Medicine (BIPS), Linzer Straße 10, 28359 Bremen, Germany

## Abstract

**Background:**

Our aim is to investigate the ability of neural networks to model different two-locus disease models. We conduct a simulation study to compare neural networks with two standard methods, namely logistic regression models and multifactor dimensionality reduction. One hundred data sets are generated for each of six two-locus disease models, which are considered in a low and in a high risk scenario. Two models represent independence, one is a multiplicative model, and three models are epistatic. For each data set, six neural networks (with up to five hidden neurons) and five logistic regression models (the null model, three main effect models, and the full model) with two different codings for the genotype information are fitted. Additionally, the multifactor dimensionality reduction approach is applied.

**Results:**

The results show that neural networks are more successful in modeling the structure of the underlying disease model than logistic regression models in most of the investigated situations. In our simulation study, neither logistic regression nor multifactor dimensionality reduction are able to correctly identify biological interaction.

**Conclusions:**

Neural networks are a promising tool to handle complex data situations. However, further research is necessary concerning the interpretation of their parameters.

## Background

The investigation of complex diseases plays an important role in genetic epidemiology where the identification of genetic risk factors is of great interest. Besides the study of main effects, the interplay of two or more genetic risk factors gains more and more attention. The identification of such a biological interaction or epistasis, however, is linked to new challenges for statistical methods. A major problem is the discrepancy between statistical and biological interaction. Statistical interaction is commonly defined as the deviation from an additive effect of single risk factors on the outcome, respectively on the transformed outcome. In logistic regression models, for example, a multiplicative structural model is applied and an additive effect on the logit-transformed outcome implies a multiplicative effect on the untransformed outcome. Therefore, statistical interaction in a logistic regression model is understood as deviation from a multiplicative effect.

On the contrary, biological interaction is present if one gene is influencing the effect of another one [1]. Both terms do not coincide as was shown for example by North et al. [2] or Foraita et al. [3]. Nevertheless, a meaningful interpretation of genetic studies requires the detection of biological interaction with statistical methods (cf. [4,5]).

A variety of parametric and non-parametric methods has been proposed for modeling and detecting gene-gene interaction, e.g. support-vector machines [6], random forests [7,8], multi-factor dimensionality reduction (MDR, [9,10]), combinatorial partitioning methods [11], focused interaction testing framework [12], classification and regression trees (CART, [13]), logic regression [14], and lasso regression [15]. A useful classification is given by Musani et al. [16], who distinguish between regression-based methods, data reduction-based methods, and pattern recognition methods in their overview.

Despite the wealth of these approaches, none of the proposed methods is optimal for all two-locus disease models (see e.g. [17-19]). Consequently, there is no established method for analyzing gene-gene interactions so far [20]. Since parametric methods have problems to detect interaction in the absence of main effects and non-parametric approaches are ineffective when main effects are present [16,21], it might well be that there is no single approach appropriate for all types of biological interaction. Currently, generalized linear models, and here logistic regression models, as well as MDR are predominantly applied (see e.g. [22-27]). Another tool that has been employed in genetic epidemiology during the last 15 years is the neural network approach (see e.g. [28-32]). Neural networks are a flexible statistical tool to model any functional relationship between covariates and response variables. Therefore, they represent a promising approach to deal with the difficulties associated with modeling biological gene-gene interactions. They have as well been successfully applied for variable selection as for example with genetic programming neural networks (GPNN, [33-36]) or grammatical evolution neural networks (GENN, [37,38]). Both approaches were developed to identify an optimal network topology. Motsinger et al. [39] successfully applied GENN to simulated genome wide association data with 500,000 Single Nucleotide Polymorphisms (SNPs) showing the general ability of neural networks to handle such large data sets. However, variable selection is not the focus of this paper.

The aim of this paper is to explore the ability of neural networks to model different types of biological gene-gene interactions. For this purpose, a simulation study is conducted to investigate the behavior of neural networks in various situations. We assume a case-control study with equal numbers of cases and controls. Following the scenarios of Risch [40] and the concept of epistatic models as classified by Li and Reich [41], different theoretical types of gene-gene interactions are studied. There are exactly two loci involved, i.e. variable selection is not a problem. The results are compared with those of logistic regression models and those of MDR analyses. Finally, the advantages and disadvantages of using a neural network approach are discussed.

## Methods

### Neural networks

A feed-forward multilayer perceptron (MLP) is chosen as neural network [42]. The general idea of an MLP is to approximate arbitrary functional relationships between covariates and response variables.

**Figure 1:**
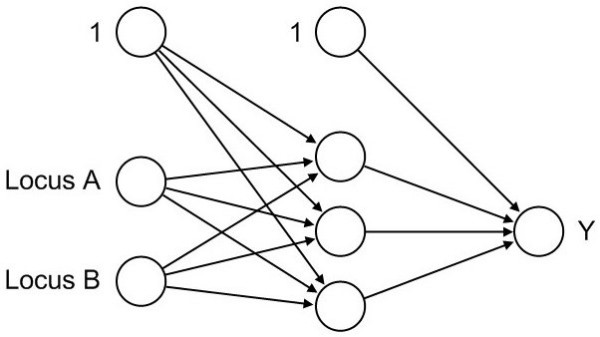
**Neural network**. Neural network with one hidden layer consisting of three hidden neurons.

The underlying structure of an MLP is a weighted, directed graph, whose vertices are called neurons and whose edges are called synapses. The neurons are organized in layers and each layer is fully connected by synapses to the next layer. The input layer contains all considered covariates and the output layer the response variables. An arbitrary number of so-called hidden layers can be included between the input and the output layer. See Figure 1 for an example of a neural network with one hidden layer.

Data is passing the neural network as signals. These signals travel the synapses and pass the neurons where the signals are processed. All incoming signals are added and the activation function *σ *is applied to the resulting sum. Additionally, a weight is attached to each of the synapses. A positive weight indicates an amplifying, a negative weight a repressing effect on the signal. During the training process, the weights are modified by a learning algorithm. The learning algorithm minimizes an error function that depends on the difference between the given output and the output estimated by the neural network. In general, the strength of the modification depends on a specified learning rate.

The minimal MLP without hidden layer is equivalent to the generalized linear model [43] and computes the function

where **w **denotes the weight vector including intercept, **x **the input vector, and *σ *the activation function. Any arbitrary function can be chosen as activation function, although most learning algorithms require a differentiable activation function. Choosing the inverse of the link function used for the logistic regression model *σ *(*z*) = 1/(1 + exp(-*z*)), the MLP without hidden layer is algebraically equivalent to the logistic regression model and computes

In this case, all weights *w*_*i *_of the MLP correspond to the regression coefficients *β*_*i *_of the logistic regression model.

Hidden layers can be included to increase the modeling flexibility. An MLP with one hidden layer computes the following function

and is capable to model any piecewise continuous function [44]. Here, there is a lack of interpretation of the parameters.

In the present paper, we investigate MLPs with at most one hidden layer. Resilient backpropagation [45] and cross entropy are chosen as learning algorithm and error function, respectively. The latter choice guarantees equivalence of the trained weights to maximum-likelihood estimation (see e.g. [46]). The employment of resilient backpropagation as learning algorithm does not require a transformation of continuous data. It solves the problem of choosing an appropriate learning rate for each data situation.

### Design of the simulation study

We conduct a simulation study, where neural network models are used to fit different two-locus disease models in a case-control design. For each of these models, one low risk and one high risk scenario is simulated. Unconditional logistic regression models are fitted to the same data sets to compare the results with an established method. For judging the ability to model the underlying disease model, the estimated penetrance matrices are compared to the theoretical penetrance matrices.

### Two-locus disease models

Six different two-locus disease models are considered: three models introduced by Risch [40] and three different epistatic models. They can be distinguished by the structure of their penetrance matrices *f *= [*f*_*ij*_]_*i*, *j*_, where *i*, *j *∈ {0, 1, 2} represent the genotype at the two loci.

1. The first two-locus disease model is Risch's additivity model (ADD). Here, the penetrance matrix is given by summing the so-called penetrance terms *a*_*i *_and *b*_*j*_

where *Y *denotes the case-control status and *G*_*A *_and *G*_*B*_, *G*_*A*_, *G*_*B *_∈ {0, 1, 2}, the genotypes at the two involved loci. The penetrance terms *a*_*i *_and *b*_*j *_are restricted to 0 ≤ *a*_*i*_, *b*_*j *_≤ 1 and *a*_*i *_+ *b*_*j *_≤ 1. This model represents biological independence of both loci.

2. For Risch's heterogeneity model (HET), the penetrance matrix is also determined by the penetrance terms

Like the additivity model, the heterogeneity model describes a model of biological independence for 0 ≤ *a*_*i*_, *b*_*j *_≤ 1. However, in this case no further constraints on the penetrance terms are necessary.

3. The third setting is Risch's multiplicative model (MULT). The penetrance matrix is given by the penetrance terms as follows

The multiplicative model represents biological interaction.

4. In the first epistatic model (EPI RR), the penetrance matrix is given by a matrix of the following type:

where the constant term *c *denotes the baseline risk of getting the disease and *r *the risk increase or decrease. This model assumes that both genes have a recessive effect on the disease, since there is only an increased or decreased risk if both loci carry two mutated alleles.

5. The penetrance matrix of the second epistatic model (EPI DD) is as follows

i.e. both loci are assumed to be dominant. In this setting, an increased or decreased risk is only observed if both loci carry at least one mutated allele.

6. The last considered scenario is a mixed epistatic model (EPI RD). The penetrance matrix is given by

In this situation, one gene (*A*) has a recessive and one gene (*B*) has a dominant effect on the disease.

All epistatic models represent gene-gene interaction. By choosing the parameters *r*, *r*_1_, *r*_2 _and the ratios *a*_1_/*a*_0_, *a*_2_/*a*_0_, *b*_1_/*b*_0_, and *b*_2_/*b*_0_, respectively, different risk scenarios can be generated.

### Data generation

The data generation follows a two-step procedure. As a first step, basic populations with one million observations are simulated. For the six two-locus disease models introduced above we investigate two risk scenarios each (see Table 1). This results in 12 basic populations with two biallelic loci, *A *and *B*. The genetic information is drawn randomly with a minor allele frequency for both loci of 0.3 to ensure sufficient cell frequencies in the final case-control samples. Both loci are assumed to be in linkage equilibrium and it is assumed that the Hardy-Weinberg equilibrium holds. The case-control status is drawn according to probabilities of a given penetrance matrix in relation to the respective disease model and the risk scenario. In all 12 settings, parameters are chosen such that the overall disease prevalence is equal to 0.01. The genotype information is described by a codominant coding, i.e. the genotype at each locus represents the number of mutated alleles.

**Table 1:** Risk scenarios.

Two-locus disease model	Low risk scenario	High risk scenario
ADD, HET, MULT	*a*_1 _= 2·*a*_0_	*a*_1 _= 5·*a*_0_
	*a*_2 _= 4·*a*_0_	*a*_2 _= 10·*a*_0_
	*b*_1 _= 5·*b*_0_	*b*_1 _= 5·*b*_0_
	*b*_2 _= 10·*b*_0_	*b*_2 _= 10·*b*_0_

EPI RR	*r *= 5	*r *= 10

EPI DD, EPI RD	*r*_1 _= 2	*r*_1 _= 5
	*r*_2 _= 4	*r*_2 _= 10

Applied risk scenarios for all two-locus disease models.

As a second step, 100 case-control samples with 1,000 cases and 1,000 controls are drawn randomly from each basic population, i.e. each combination of two-locus disease model and risk scenario. Overall, this results in 12 times 100 case-control samples that will be analyzed.

### Modeling the data

Model-building with neural networks is done using six different network topologies from zero neurons in the hidden layer (i.e. no hidden layer) up to five neurons in the hidden layer. Each topology is trained five times with synaptic weights initialized with random numbers drawn from a standard normal distribution to avoid local minima. From these fitted models, the best model for each data set, i.e. the network topology, is chosen using Akaike's Information Criterion (AIC, [47]).

The following five logistic regression models are fitted to each data set: the null model (NM), three main effect models (only locus A (SiA), only locus B (SiB), both main effects (ME)), and a full model including both main effects and an interaction term (FM). The best model for each data set is chosen based on the AIC. Note that the neural network with zero neurons in the hidden layer is algebraically equivalent to the main effect model ME. In a second approach, logistic regression models are fitted to the data with two dichotomous design variables representing each locus. Instead of counting the number of mutated alleles, these two variables reflect the heterozygous genotype and the homozygous genotype with two mutated alleles, respectively. For instance, the main effect model for locus *A *only (SiA) is modeled with a codominant coding as

as opposed to

with design variables. The observation is indexed by *k*, *β *represents the regression coefficients and **1 **an indicator function. Table 2 gives an overview of the fitted statistical models and the numbers of needed parameters for all considered models.

**Table 2:** Number of parameters.

	Neural network	
0 hidden neurons	3	
1 hidden neuron	5	
2 hidden neurons	9	
3 hidden neurons	13	
4 hidden neurons	17	
5 hidden neurons	21	

	**Logistic regression**	**Logistic regression (DV)**

Null model (NM)	1	1
One main effect (SiA/SiB)	2	3
Both main effects (ME)	3	5
Full model (FM)	4	9

Number of parameters for neural networks, logistic regression models and logistic regression models with design variables (DV).

These three applied statistical methods deliver as output an estimation of the probability to be a case, i.e. the penetrance for each genotype-genotype combination. We compare these estimated penetrance matrices to the theoretical ones to judge the ability of the statistical methods to model the underlying two-locus disease model. A penetrance matrix derived from a case-control sample differs considerably from one derived from the basic population, since the penetrance matrix depends on the prevalence of disease in the considered data. Therefore, we have to compute the theoretical penetrance matrix for the case-control sample using the penetrance matrix from the basic population, the allele frequencies and the prevalence of the population (see appendix for an example). The comparison of the obtained theoretical penetrance matrix with the penetrance matrices estimated by the three different statistical approaches gives results which are independent from sampling error, since the theoretical penetrance matrix symbolizes a perfectly drawn case-control sample. For each of the 12 populations, the mean absolute difference between theoretical and estimated penetrance matrix is calculated element by element for each genotype-genotype combination over the *n *= 100 case-control samples:

where *i*, *j *∈ {0, 1, 2}, and *f*_*ij *_and  denote the entries of the theoretical and estimated penetrance matrix of the *k*th sample, respectively. Furthermore, the sum of the mean absolute differences ∑_*i*, *j*_*E*_*ij *_is considered.

The data generation and the statistical analyses for neural network and logistic regression are performed using R [48]. The package for the MLP, neuralnet, was newly implemented by our group and is published on CRAN [49].

Additionally, the MDR approach is applied to the data. The analyses are conducted by the java-based open source software MDR release 1.2.5 with default configurations [50]. In particular, analysis configurations are specified as follows: the random seed is set to zero, the attribute count maximum is set to two and the cross-validation count to ten. The MDR identifies a set of functional variables that is best for classifying cases and controls. Due to the number of simulated loci, the software can only select one of three sets: either locus *A *or locus *B *only or both loci. Additionally, it provides a dendrogram to distinguish between redundant and synergistic variables based on information theory [51].

## Results

In a first step, we investigate the ability of neural networks and logistic regression models to model different two-locus disease models. Table 3 shows the results for Risch's additivity model. Here, the sum of the mean absolute differences between estimated penetrance and theoretical penetrance matrix is lowest for the neural networks. This is most pronounced in the high risk scenario (∑*E*_*ij *_= 0.2059 for neural networks versus ∑*E*_*ij *_= 0.2544 and ∑*E*_*ij *_= 0.2804 for logistic regression models without and with design variables). Logistic regression models with design variables have in general higher deviations than those without design variables. These results are also reflected in the element-wise comparison of the estimated matrices. For each of the risk scenarios, the neural network estimates five out of nine penetrances with the highest accuracy, i.e. with smallest difference to the theoretical penetrance, compared to the logistic regression models. The heterogeneity model yields virtually the same results as the additivity model (results not shown).

**Table 3:** Additive model (ADD).

	Low risk	High risk
	*a*_1 _= 2·*a*_0_; *a*_2 _= 4·*a*_0_	*a*_1 _= 5·*a*_0_; *a*_2 _= 10·*a*_0_
	*b*_1 _= 5·*b*_0_; *b*_2 _= 10·*b*_0_	*b*_1 _= 5·*b*_0_; *b*_2 _= 10·*b*_0_
**Theoretical penetrance matrix**		

**Neural network**		
Mean absolute difference *E*		
Sum	0.2313	0.2059

**Logistic regression**		
Mean absolute difference *E*		
Sum	0.2530	0.2544

**Logistic regression (design variables)**		
Mean absolute difference *E*		
Sum	0.2897	0.2804

Mean absolute differences between theoretical and estimated penetrance matrices from 100 replications in the low and high risk scenario.

For Risch's multiplicative model (see Table 4), the logistic regression models with design variables have the best fit to the underlying data as is reflected by the lowest mean absolute difference of the estimated to the theoretical penetrance matrix (∑*E*_*ij *_= 0.1637 resp. ∑*E*_*ij *_= 0.1833 for the two risk scenarios). This holds true for the sum as well as for the single entries in both risk scenarios. Although neural networks show worse accuracy for both risk scenarios (∑*E*_*ij *_= 0.2428 resp. ∑*E*_*ij *_= 0.2178), they mostly need two neurons in the hidden layer (results not shown), that is nine parameters as opposed to five parameters that are used most often in the logistic regression models with design variables. This implies that the higher degrees of freedom do not lead to a better fit in the situation of a multiplicative model. Furthermore, logistic regression models without design variables are not able to model this disease model (∑*E*_*ij *_= 0.3965 resp. ∑*E*_*ij *_= 0.4887).

**Table 4:** Multiplicative model (MULT).

	Low risk	High risk
	*a*_1 _= 2·*a*_0_; *a*_2 _= 4·*a*_0_	*a*_1 _= 5·*a*_0_; *a*_2 _= 10·*a*_0_
	*b*_1 _= 5·*b*_0_; *b*_2 _= 10·*b*_0_	*b*_1 _= 5·*b*_0_; *b*_2 _= 10·*b*_0_
**Theoretical penetrance matrix**		

**Neural network**		
Mean absolute difference *E*		
Sum	0.2428	0.2178

**Logistic regression**		
Mean absolute difference *E*		
Sum	0.3965	0.4887

**Logistic regression (design variables)**		
Mean absolute difference *E*		
Sum	0.1637	0.1833

Mean absolute differences between theoretical and estimated penetrance matrices from 100 replications in the low and high risk scenario.

The results for the epistatic models are presented in Tables 5, 6 and 7. In the first epistatic model, the mean absolute differences between the theoretical penetrance matrices and the estimated penetrance matrices of the neural networks are generally lower (sum and single entries) than those of the logistic regression models (see Table 5). In particular, the logistic regression model without design variables performs poorly in the high risk scenario (∑*E*_*ij *_= 0.6150 for logistic regression models without design variables versus ∑*E*_*ij *_= 0.1410 for neural networks).

**Table 5:** Epistatic model - recessive (EPI RR).

	Low risk	High risk
	*r *= 5	*r *= 10
**Theoretical penetrance matrix**		

**Neural network**		
Mean absolute difference *E*		
Sum	0.2071	0.1410

**Logistic regression**		
Mean absolute difference *E*		
Sum	0.4849	0.6150

**Logistic regression (design variables)**		
Mean absolute difference *E*		
Sum	0.3503	0.2755

Mean absolute differences between theoretical and estimated penetrance matrices from 100 replications in the low and high risk scenario.

**Table 6:** Epistatic model - dominant (EPI DD).

	Low risk	High risk
	*r*_1 _= 2; *r*_2 _= 4	*r*_1 _= 5; *r*_2 _= 10
**Theoretical penetrance matrix**		

**Neural network**		
Mean absolute difference *E*		
Sum	0.3095	0.2524

**Logistic regression**		
Mean absolute difference *E*		
Sum	0.3132	0.6528

**Logistic regression (design variables)**		
Mean absolute difference *E*		
Sum	0.3071	0.2648

Mean absolute differences between theoretical and estimated penetrance matrices from 100 replications in the low and high risk scenario.

**Table 7:** Epistatic model - mixed (EPI RD).

	Low risk	High risk
	*r*_1 _= 2; *r*_2 _= 4	*r*_1 _= 5; *r*_2 _= 10
**Theoretical penetrance matrix**		

**Neural network**		
Mean absolute difference *E*		
Sum	0.2239	0.1563

**Logistic regression**		
Mean absolute difference *E*		
Sum	0.5105	0.8658

**Logistic regression (design variables)**		
Mean absolute difference *E*		
Sum	0.2799	0.2329

Mean absolute differences between theoretical and estimated penetrance matrices from 100 replications in the low and high risk scenario.

The results for the epistatic model with two dominant loci are different for the two risk scenarios (see Table 6). In the low risk scenario, none of the three statistical approaches is able to satisfactorily estimate the theoretical penetrance matrix of the disease model. The sum of the mean absolute differences ranges from ∑*E*_*ij *_= 0.3071 to ∑*E*_*ij *_= 0.3132 for the three approaches. In the high risk scenario, neural networks slightly outperform the logistic regression models with design variables, whereas the regression models without design variables completely fail to detect the characteristic structure of the underlying penetrance matrix (∑*E*_*ij *_= 0.2524 for neural networks versus ∑*E*_*ij *_= 0.2648 and ∑*E*_*ij *_= 0.6528 for logistic regression models with respectively without design variables). The better fit of neural networks and logistic regression models with design variables is traded off by a high number of parameters: both approaches need on average about 9 parameters (results not shown).

The structure of the theoretical penetrance matrices given by the mixed epistatic model with one dominant and one recessive locus is again best modeled by neural networks (see Table 7). This can be observed for the sum and for the single entries of the mean absolute differences between the theoretical and the estimated penetrance matrices in both risk scenarios. The logistic regression models without design variables are again not able to identify this structure. The mean absolute differences are much higher as opposed to the differences of the other approaches (e.g ∑*E*_*ij *_= 0.8658 and ∑*E*_*ij *_= 0.2329 for logistic regression models without respectively with design variables and ∑*E*_*ij *_= 0.1563 for neural networks in the high risk scenario).

In a second step, we investigate whether the standard methods logistic regression and MDR are able to detect the interaction given by the four two-locus disease models representing biological interaction. Table 8 summarizes the results of the logistic regression models with and without design variables regarding the selected models for each population. The bold numbers mark the mode of the selected models. In the upper part of the table, the two-locus disease model (ADD, HET) agrees with the statistical model when a statistical model of independence (NM, SiA, SiB, ME) is selected. In the lower part of the table, the two-locus disease model representing biological interaction (MULT, EPI RR, EPI DD, EPI RD) agrees with the statistical model when the full model (FM) is selected. Both logistic regression models yield similar results for the additivity and the heterogeneity model. In most cases, interaction terms are included into the statistical models despite the fact that the underlying data follows a disease model representing independence. This is especially true in the high risk scenario. In the low risk scenario there is one notable exception for the heterogeneity model: in more than half of the replications, the logistic regression models with design variables contain no interaction term.

**Table 8:** Selected logistic regression models (LRM).

		LRM with design variables					
		Statistical model (# parameters)					
**Two-locus disease model**	**Risk scenario**	**NM (1)**	**SiA (3)**	**SiB (3)**	**ME (5)**	**FM (9)**	∑

**ADD**	low			1	39	**60**	100
	high				7	**93**	100
**HET**	low				**55**	45	100
	high				10	**90**	100

**MULT**	low				**90**	10	100
	high				**88**	12	100
**EPI RR**	low			6		**94**	100
	high					**100**	100
**EPI DD**	low				3	**97**	100
	high					**100**	100
**EPI RD**	low		**61**		19	20	100
	high		**57**		14	29	100

		**LRM without design variables**					
		**Statistical model (# parameters)**					
		**NM (1)**	**SiA (2)**	**SiB (2)**	**ME (3)**	**FM (4)**	∑

**ADD**	low			1	27	**72**	100
	high				4	**96**	100
**HET**	low				30	**70**	100
	high				6	**94**	100

**MULT**	low				**72**	28	100
	high				**54**	46	100
**EPI RD**	low	7	6	9	3	**75**	100
	high					**100**	100
**EPI DD**	low				2	**98**	100
	high					**100**	100
**EPI RD**	low		**60**		19	21	100
	high		38		23	**39**	100

In the upper part of the table, the two-locus disease model (ADD, HET) agrees with the statistical model when a statistical model of independence (NM, SiA, SiB, ME) is selected. In the lower part of the table, the two-locus disease model representing biological interaction (MULT, EPI RR, EPI DD, EPI RD) agrees with the statistical model when the full model (FM) is selected. Bold numbers mark the mode of the selected models in the low and high risk scenario.

Different two-locus disease models representing gene-gene interaction lead to varying results when logistic regression models are applied. The logistic regression models do not include an interaction term in most replications when the multiplicative model is the underlying disease model. That means that the logistic regression models fail to detect the underlying biological interaction. The recessive and the dominant epistatic model are correctly represented by the full model in most situations. Only in the low risk scenario of the recessive epistatic model, the logistic regression models without design variables choose a broad variety of models in a quarter of the replications. For the mixed epistatic models, the logistic regression models perform poorly: Since model SiA is mostly selected, the main effect for the (dominant) locus *B *is not detected in more than half of the replications and the interaction effect is included only in about 20% of the replications.

Table 9 summarizes the results for the MDR analyses. It shows the selected variables for each population in combination with their identification as synergistic or redundant. Bold numbers again mark the mode of selected sets in each population. Even though both main effects are present in all populations, the MDR approach often selects a set consisting of only one locus independent of whether the underlying two-locus disease model represents independent effects or biological interaction. This holds true for the additive and the heterogeneity model in the low risk scenario, where only locus *B *is selected for most of the 100 data sets, and the mixed epistatic model, where a set consisting of locus *A *only is mainly selected. Apart from the mixed epistatic model, both variables are selected correctly for the disease models representing biological interaction. As for the logistic regression model, the sets of selected variables strongly vary for the recessive epistatic model.

**Table 9:** MDR analyses: selected variables and identification as redundant or synergistic behavior.

		MDR analyses						
		Redundant			Synergistic			
Two-locus disease model	Risk scenario	Only A	Only B	Both	Only A	Only B	Both	∑
**ADD**	low		**82**	18				100
	high		7	**93**				100
**HET**	low		**68**	32				100
	high	1	6	**93**				100

**MULT**	low		7	**93**				100
	high			**100**				100
**EPI RR**	low	10	22	**39**	2	4	23	100
	high	18	17	**59**	1	2	3	100
**EPI DD**	low			12		1	**87**	100
	high			18			**82**	100
**EPI RD**	low	**63**		34			3	100
	high	**97**		3				100

Bold numbers mark the mode of the selected variables in the low and high risk scenario.

Additionally, the provided dendrogram can be applied to distinguish between redundancy and synergism. These concepts are related to independence and interaction in our context [52]. Both loci are categorized as redundant for most of the investigated populations. Only the dominant epistatic model is correctly identified as a synergistic model for the majority of the data sets.

No similar statement about the agreement of disease and statistical model can be made for neural networks since there is no equivalent to the concept of interaction terms. Neural networks with one or two neurons in the hidden layer (i.e. models with five or nine parameters) are the most frequent models selected in the simulation study.

## Discussion

In our simulation study, we investigated whether neural networks are able to model different types of gene-gene interaction in case-control data. For this purpose, we analyzed simulated data of six different two-locus disease models in two different risk scenarios with neural networks and compared the results to logistic regression models using two different approaches for coding the genotype information. Additionally, we investigated whether logistic regression models or the MDR approach, which are two widely used methods in applications, are suitable to identify biological interaction.

For the majority of the investigated situations, the theoretical penetrance matrix is estimated most accurately by neural networks as opposed to logistic regression models. The exception is the multiplicative model in both risk scenarios and the dominant epistatic model in the low risk scenario. Although, in these situations, neural networks use two neurons in the hidden layer, i.e. nine parameters, in most replications, they are not able to exploit the flexibility to correctly represent this disease model. For the logistic regression models, it can be stated that the disease models of independence are better represented by a logistic regression model without design variables and the disease models of interaction are better represented by a logistic regression model with design variables. In situations where interaction is present using a logistic regression model without design variables might lead to wrong results. Since the underlying disease model is usually not known beforehand, no recommendation can be given whether to employ design variables or not. Both logistic regression models mostly select a main effect model to represent the multiplicative model. The inclusion of interaction terms signifies deviations from the structural model rather than from the disease model representing independence. Consequently, the underlying biological interaction represented by the multiplicative and the epistatic models cannot be read off the fitted logistic regression models. The same holds true for the MDR approach. It is not possible to correctly identify biological interaction based on the sets of selected variables or based on the dendrograms since the additive and the heterogeneity model as independence models cannot be distinguished from the four models representing biological interaction with neither of these two criteria.

The results confirm previous studies that demonstrate the excellent modeling capacities of neural networks [32]. We investigated, whether the weaker performance of the neural network especially for the multiplicative model might be due to a wrong model selection criterion. Alternatively to the AIC, we calculated Bayes Information Criterion (BIC, see [53]) for all models (results not shown). However, employing the BIC for model selection does not improve the performance of the neural network as opposed to the logistic regression models. In fact, the stronger performance of the logistic regression model is supposed to be due to the fact that the multiplicative model exactly corresponds to the structural model of the logistic regression model.

It might be disputed whether the applied risk scenarios feature too large genotype relative risks to be meaningful for real-data applications. For the recessive epistatic model as the most extreme situation, alternative scenarios were investigated employing smaller risks. All investigated approaches have difficulties detecting these smaller risks. For the logistic regression models, the null model is mostly chosen, thus, neglecting the elevated penetrance when both loci carry two mutated alleles.

Neural networks do not explicitly use interaction terms for modeling data. Unlike in logistic regression models, where an interaction term might become significant or not, there is no easy way to assess whether interaction is present using a neural network. Moreover, in models with one or more hidden layers there is no direct interpretation of the estimated parameters and the MLP is generally considered as a black-box approach. This can be seen as the biggest drawback when employing neural networks for data analyses where interpretation is a major concern. However, the modeling capacities of a neural network allow to adjust to practically any given data structure, including any interaction structure, which makes it an extremely powerful statistical tool. This advantage might even be more pronounced when modeling continuous variables, for example when modeling gene-environment interactions.

The use of neural networks in applications is currently still limited because of existing research gaps. Especially, the interpretability of the estimated weights is not yet given. Nevertheless, they offer a promising tool for exploratory analyses in candidate gene studies. For instance, they can well be applied when one is interested in odds ratios for single SNPs. The estimated odds ratios are more realistic than those estimated by logistic regression models in a lot of situations since the estimated output of neural networks better represents the underlying population. As initially stated, we did not explore the ability of neural networks for variable selection, which is a key problem in genome-wide association (GWA) studies.

## Conclusions

We explored the ability of neural networks to model different types of biological gene-gene interactions and compared them to logistic regression models and the MDR approach. The latter methods do not allow reading off the underlying two-locus disease models. Neural networks do not explicitly include an interaction term but they are able to model any data structure. Even though the estimated weights are not interpretable, this makes them a powerful statistical tool. Further research should be devoted to develop a framework for interpreting the parameters estimated by a neural network to allow a broader use of these tools.

## Authors' contributions

FG planned and carried out the simulation study and drafted the manuscript. NW drafted the manuscript. KB planned the simulation study and drafted the manuscript. All authors read and approved the final manuscript.

## Appendix

To illustrate the calculation of the theoretical penetrance matrix, we consider the epistatic model with two recessive loci. We assume that the two considered loci are in linkage equilibrium, i.e. they are marginal independent, and that the Hardy-Weinberg equilibrium holds. In the population, the probabilities are denoted as follows

This enables us to express the conditional probabilities of the genotypes given the case-control status as:

and

where  and . These conditional probabilities remain the same when drawing a case-control sample

where *P*^*s *^indicates a probability in a case-control sample. There are only changes in the joint probabilities of the genotypes *P*^*s*^(*G*_*A *_= *i, G*_*B *_= *j*) because of the change of prevalence: *P*^*s*^(*Y *= 1) = *P*^*s*^(*Y *= 0) = 0.5.

The joint probabilities can be calculated as

The theoretical penetrance matrix of the sample can now be calculated as:

For example, for the low risk scenario (*r *= 5) and an overall prevalence in the population of *K *= 0.01, the constant *c *can be calculated as *c *= 0.009686 and the theoretical penetrance matrix of the sample results in

This theoretical penetrance matrix of the sample is compared to the predicted penetrance matrices generated by the different models to judge the ability of neural networks and logistic regression models to model different two-locus disease models.
